# Refractory chylothorax after severe vomiting and coughing in a 4-year-old child

**DOI:** 10.1093/jscr/rjad466

**Published:** 2023-08-17

**Authors:** Vincent De Pauw, Siel Daelemans, Leontien Depoorter, Carola Brussaard, Dirk Smets

**Affiliations:** Thoracic Surgery Department, UZ BRUSSEL, Jette, Belgium; Pediatric Department, UZ BRUSSEL, Jette, Belgium; Pediatric Department, UZ BRUSSEL, Jette, Belgium; Radiology Department, UZ BRUSSEL, Jette, Belgium; Thoracic Surgery Department, UZ BRUSSEL, Jette, Belgium

## Abstract

Chylothorax is the accumulation of lymphatic fluid in the pleural space. It is a rare condition with potentially life-threatening disorders. In children, the etiology of chylothorax can be mainly attributed to idiopathic factors, congenital, miscellaneous, trauma and malignancies. Conservative treatments can solve most chylothorax, but refractory chylothorax can be challenging to manage. We herein present the case of a 4-year-old girl with no previous medical history who was admitted to our institution after severe vomiting and right chylothorax. The etiological assessment could not identify specific causes. Initial treatment was conservative but after 14 days, the patient showed no improvement. An exploratory thoracoscopy using indocyanine green showed no active leaks. Pleurodesis was performed and, later on, ligation of the ductus thoracicus. Hereafter, the patient progressed favorably. Even though conservative treatments of chylothorax show a high success rate, the efficacy of additional therapies and the benefits between surgical procedures need further investigation.

## INTRODUCTION

Chylothorax is an effusion of chyle into the pleural cavity. It is a rare condition with potentially life-threatening disorders that may cause profound adverse effects. In young children, the etiology of chylothorax can be mainly attributed to idiopathic factors, congenital, miscellaneous, trauma (iatrogenic or not) and malignancies [1, 2]. Conservative treatments can solve most chylothoraxes, but refractory chylothoraxes can be challenging to manage.

## CASE REPORT

We herein present the case of a 4-year-old girl with no previous medical history who was admitted to the emergency department for a severe cough associated with persistent vomiting. Computed chest tomography showed massive right pleuritis with total lung collapse. There were no additional abnormalities. A chest tube was inserted. The milky-like aspect and the high triglyceride level (TG = 733 mg/dl) in the pleural fluid confirmed the diagnosis of chylothorax.

**Figure 1:**
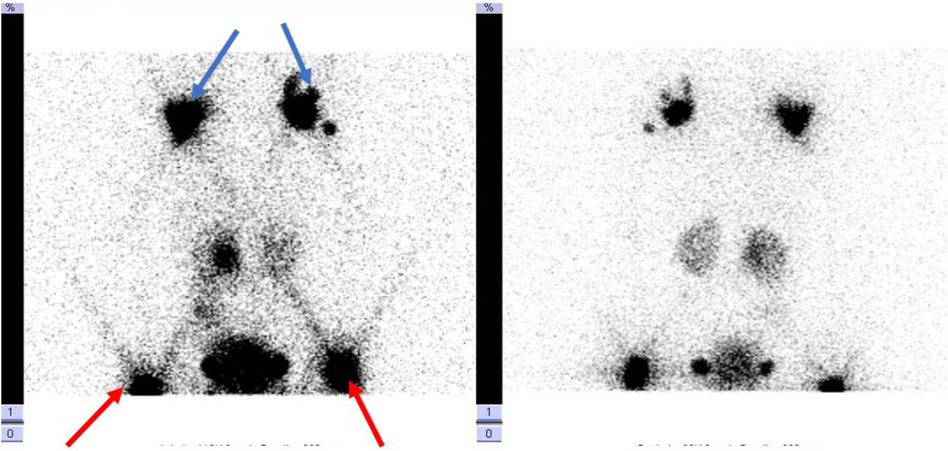
Lymphoscintigraphy: blue arrow indicates axillary lymph nodes, the red arrow shows the inguinal lymph nodes. They are no signs of tracers in the thorax cavity.

**Figure 2:**
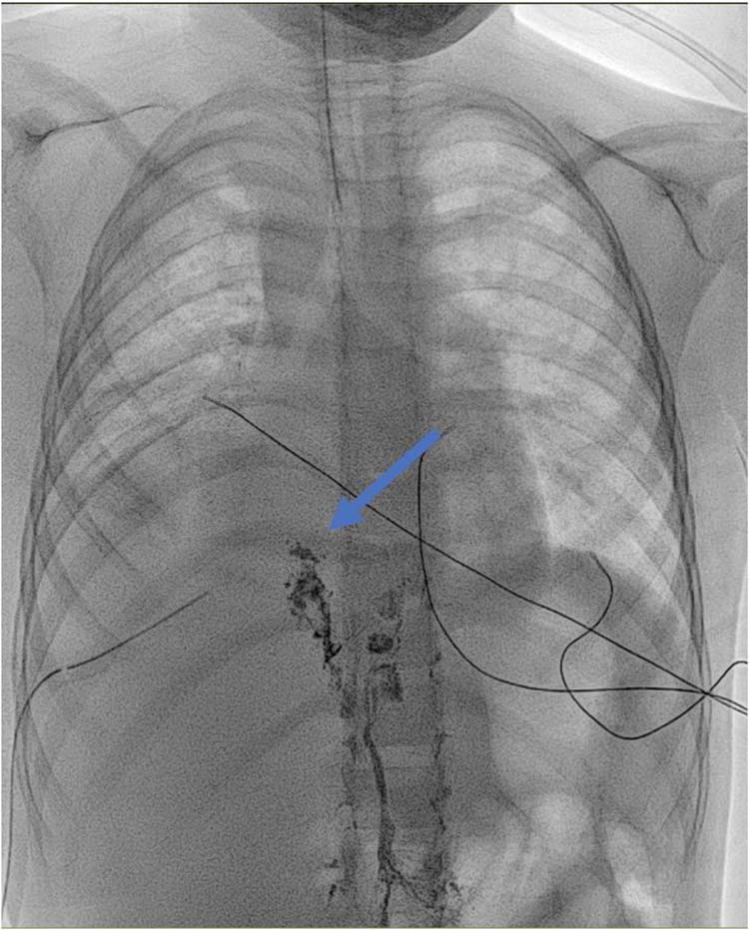
Lymphangiography: detection of lipiodol in the ductus thoracicus (blue arrow). The marker stops at the clipping of the ductus thoracicus. No leakage is detected.

Antibiotic therapy and parenteral nutrition were initiated. In addition, Sandostatin was started at a dose of 1mcg/kg/h. Primary workup lymphoscintigraphy showed no lymphatic leakage or lymphatic abnormalities (Fig. 1). Examinations addressing malignancy and other causes of chylothorax did not reveal any additional pathologic findings. After 14 days of conservative treatment, the patient showed no significant improvement with a pleural fluid production of over 500 cc/24 h. Therefore, it was decided to perform an exploratory thoracoscopy with the injection of indocyanine green. The thoracic duct was visualized during the procedure but showed no active leaks. A pleurodesis was performed. After surgery, there was an improvement with decreased pleural fluid production. Medium Chain Triglyceride (MCT) diet was introduced, and somatostatin was slowly reduced. Unfortunately, after 4 days, the patient reacquired a significant amount of pleural fluid. The thoracic duct was clipped, and a lung biopsy was performed due to the lack of improvement. Clinically, the patient progressed inconsistently with a further episode of vomiting, which resolved spontaneously. The thoracic tube was removed 10 days after the second intervention. Follow-up radiographs showed the return of pleural effusion with the incomplete collapse of the lower lobe. A lymphangiography confirmed the excellent positioning of the clip and could not find any other abnormalities or leaks (Fig. 2). The genetic and anatomopathological study did not show any irregularities. Four months after discharge, the overall condition of the patient was good.

## DISCUSSION

Chylothorax’s etiology can be divided into various categories: idiopathic, congenital, traumatic, high central venous pressure, malignancy or miscellaneous. Cardiothoracic surgery is the leading cause in infants, with idiopathic and lymphoma accounting for 55.6, 40.7 and 3.8%, respectively [1]. Apart from the frequently encountered causes, other rarer etiologies have also been described. It has been reported that an increase in intrathoracic pressure can cause laceration of organs such as the esophagus and trachea, resulting in Boerhaave’s syndrome, Mallory Weiss or subcutaneous emphysema. However, chylothorax has been rarely described after severe vomiting and coughing [3].

The assessment should be directed to exclude malignant, obstructive, infectious, cardiac or genetic pathologies for chylothorax who present without a history of trauma or surgery. The 2017 American College of Radiology Appropriateness Criteria indicate that if the etiology of the chylothorax is unknown, computed tomography (CT) associated with positron emission tomography (PET) scanning can rule out vascular abnormalities and malignancy. In addition, magnetic resonance imaging of the chest and abdomen can be added to better visualize lymphatic vessel discrepancies [4]. Lymphoscintigraphy has also shown utility for detecting abnormal lymphatic patterns, visualizing the thoracic duct anatomy and potentially detecting a lymphatic leak [5]. Although it is not recommended in practice, it offers an exciting alternative to lymphangiography with relatively equal sensitivity and specificity [6].

Management of chylothorax algorithms can be contradictory. It can be achieved either by conservative means or surgery. In symptomatic patients, a chest tube should be inserted to reduce the intrathoracic pressure, allowing lung expansion and potentially helping seal the leak by filling the pleural space. Conservative management can be attempted if the chyle flow rate is <500 ml/day with a report success rate varying between 27 and 100% [7]. This involves dietary management by avoiding enteral fat intake using an MCT diet or total parenteral nutrition. However, such a diet generally induces severe malnutrition from energy deficiency, liposoluble vitamins and essential fatty acids. Furthermore, this approach extends hospitalization from 4 to 6 weeks. The addition of somatostatin may be associated with conservative management in patients with insufficient dietary methods. It decreases portal flow and gastrointestinal secretions, significantly reducing lymphatic outpour through the thoracic duct. Therefore, it could prevent intervention in 50% of the cases. Despite the benefits seen in some studies, the factors contributing to somatostatin success have yet to be discovered [8]. In nonresponding chylothorax, surgical intervention is required but is associated with a higher risk of complications.

Nevertheless, the timing of surgery needs to be clearly defined. Some studies propose conservative management of 2–4 weeks before surgery [2]. Others consider a pleural fluid production greater than 100 ml per year of age per day as an indication for immediate intervention in children. Surgical procedures include thoracic duct ligation of adjacent leaking lymphatics, chemical pleurodesis or pleuroperitoneal shunting. For patients without evidence of thoracic duct rupture, pleurodesis is, for some authors, the treatment of choice [9]. Pleuro-peritoneal shunt remains the last option in managing chylothorax. Nevertheless, some authors described it as efficient and well tolerated and preferred this method due to its less definitive nature than pleurodesis. Lymphangiography performed with an oil-based contrast has also been described as an alternative for surgical treatment in case of lymphatic leakage with a small debit. The leakage will close due to the sclerosing properties of the contrast agent [10].

In our cases, the patient received all the appropriate treatments described above except the pleuro-peritoneal shunting with, after 8 weeks of hospitalization, there was a stabilization of the chylothorax and healthy evolution.

In conclusion, the management of chylothorax is numerous. Even though conservative treatments show a high success rate, further studies are required to fully understand the pathophysiological mechanisms around non-traumatic chylothorax, the efficacy of additional therapies such as somatostatin and the benefits between thoracic duct ligation, pleurodesis and pleuroperitoneal shunts.

## Data Availability

The data can be retrieved by emailing the corresponding author.
